# St. Luke's Hospital for the Insane

**Published:** 1890-01-18

**Authors:** 


### The Hospital Workshop.

### No. XX.—ST. LUKE'S HOSPITAL FOR THE INSANE.

(Part II.)

(by our special commissioner).

On the male side a rather incoherent old gentleman was pointed out as having been an inmate since 1848, forty-two years ago. Another patient, who had been there for forty years, hailed our appearance with a cheerful and melodious "Cuckoo!" This man (so we were told) used to help the plumber on the roof until one day he threatened to throw his fellow-worker in the street, since which occasion his services have been dispensed with in that particular part of the building. Space does not permit us to notice many cases, but passing mention may be made of several suffering from that most interesting and mysterious disease, general paralysis of the insane. As already stated, patients afflicted with that complaint are not admitted to St. Luke's, still, they are not sent away should it develop subsequently. The disease usually runs a rapid course. It is characterised by a gradual paralysis that invades the whole body until the patient drifts into a miserable condition, and can no longer stand, speak, move, or help himself in any way whatever. In the early and "excitable" stage delusions of an exaggerated and grandiose kind are usually present. For instance, one man came under the writer's observation who had formerly been an engine-driver by occupation. His pet idea was to make himself a locomotive engine of solid gold, and to build a bridge across the Atlantic so that he might run an ocean express to America. At the same time he proposed to give a brand new guinea to every inmate of the asylum. After such magnificent promises and undertakings it is not a little ludicrous to hear their proposer suddenly change his tone and beg for a halfpenny to buy tobacco. At a comparatively early period of the disease the muscles of the tongue begin to get paralysed, and this causes a peculiar and typical thickness of utterance. One of the St. Luke's patients appeared to have an interesting theory about turning the North Sea whales to some useful purpose in navigation. To-day, however, he declines to say very much upon the subject, upon the ground that the scheme must be carried out by someone who has plenty of ships and who will be able to catch and tame the whales. This man was said to be full of all sorts of picturesque fancies, and the medical superintendent was inclined to put down his unusual reticence to the fact that your commissioner was at the time entering short notes in a pocket-book. Another patient, affected with general paralysis in a more advanced stage, was seated in a chair by the fire. He was a stout man of middle age, his face blurred and vacant, and his words hardly distinguishable, as in reply to repeated questions he solemnly asserted his age to be one hundred and thirty-six, his weight to be fifteen stone, and his height sixteen feet.

At the back of the building lie the recreation grounds for men and for women, the chapel, the stables and clock-tower, billiard-rooms and tennis-lawns. Behind the "middle house" one walks into a garden, so called, but it is a place where hardy bedding plants, although protected by glass, refuse to live, and where ivy, with its evergreen robustness, finds it almost impossible to face the murky atmosphere. In addition to soot and smoke, there are some vinegar and other works that do their best to poison everything in the neighbourhood. Curiously enough, the hospital seems to be comparatively unaffected by the absence of fresh air, if we may judge from the longevity, the bills of health, and the absence of epidemics both among resident staff and among patients. The oldest inhabitant is a woman admitted forty-five years ago, and who thus beats the record of the men by three years. Some time ago a female patient died after having spent no less than fifty-six years of her life in the ward. Of course a good deal of this healthiness must be attributed to careful sanitary arrangements. Quite lately Wenham gas-burners have been inserted throughout the building; washable papers substituted for the old-fashioned kind; and the whole drainage system modernised at a cost of £600.

The plot of ground immediately behind the "middle house" is planted with the sombre and struggling ivy already alluded to, and contains a group of trees, of which the biggest is an ancient mulberry, with chained-up branches. Notwithstanding its age, which must be considerable, judging from its size and from the fact that the mulberry is of slow growth, yet the tree is reported to be prolific of fruit. When we reached the spot it was getting late in the afternoon, and a number of birds were flocking in to take up their quarters for the night. Before very long the branches were filled by a crowd of twittering and chattering sparrows. In this part of London trees available for roosting are few and far between, so that a shelter of this kind becomes—as the agents say—"an extremely valuable and eligible freehold dwelling site." Birds soon find out where they are safe, and the London sparrow, above all his species, knows how to look after himself. He is quick, quarrelsome, and shrewd as a street urchin that has to scramble, like himself, for a living in the street. A study of the habits and manners of *passer domesticus*, variety *Londinensis*, would well repay any lover of natural history. The gardens of other hospitals are frequented in the same way. Not very long ago an owl took up its quarters in the trees at Guy's Hospital, and soon thinned out the sparrows that locate themselves there in hundreds. The solemn bird of prey was recognised by a man in Soho as belonging to him, but it has hitherto resisted all attempts at capture.

The airing ground for the men includes an old detached burial ground belonging to St. Luke's Church, which has been asphalted over for its present purpose. Among the surrounding buildings are two billiard-rooms; the one which is nearest the central ground has been lately refitted, and presents a most cheerful appearance with its wood panelled walls and many other arrangements for warmth and comfort. There is nothing very noticeable about the chapel, excepting its east end, which is decorated with a handsome design in tiles and fresco. An organ appears to be wanted, and would, no doubt, prove a desirable addition.

Coming back to the ground floor of the building, we were shown some interesting relics of the time when insanity was regarded as a thing to be punished rather than as a disease to be carefully and scientifically treated. Founded on the same principle as *similia similibus curantur*, the pernicious teaching "to make the mad more mad," led our forefathers into practices that we now regard with feelings of horror and amazement. The original refractory ward is no longer tenanted, for in the light of modern ideas it is quite unfit for human habitation. It is a kind of long, low-arched crypt, lighted with windows along one side. Let into the piers of the arches are little blocks of wood, to which were formerly fastened the chains that held the patients. The only accommodation afforded them appears to have been some straw litter thrown on the stone floor, and the poor wretches were thus left to drag out their miserable existence. At the further end is a dark room intended for a punishment cell, and which is constructed out of the solid masonry. Another relic of the bad old times is a huge wooden armchair, in which the unfortunate patient was seated and fastened down by a board locked across the arms. Once inside this contrivance, the patient was helpless, and, if he became violent, the sides and back of the chair were padded so as to keep him from injury. As may be readily imagined, treatment after this unenlightened fashion did not yield very satisfactory results. On the other hand, under modern methods of non-restraint, mental occupation, good food, amusement, plentiful exercise, and careful attention to general health, the large percentage of forty recoveries in a hundred is claimed. There is little reasonable doubt that even better results might be obtained if all cases were brought under skilled treatment at an early stage. Unfortunately, the favourable time is too often allowed to slip by until the damage is irretrievable. Besides which, there is a popular prejudice against asylums which attaches a certain amount of reproach to residence in one of those institutions. Some day, it is to be hoped, people generally will learn to look upon insanity as a disease to be carefully tended, more especially in its early stages. St. Luke's shows in an interesting manner how a humane present can be grafted on the austere stock of a mistaken and unscientific past.

